# First dietary study of the critically endangered black morph Bornean banded langur (*Presbytischrysomelaschrysomelas*) using DNA metabarcoding

**DOI:** 10.3897/BDJ.13.e161184

**Published:** 2025-08-06

**Authors:** Mohammad Noor-Faezah, Abd Rahman Mohd-Ridwan, Nur-Aizatul Tukiman, Roberta Chaya Tawie Tingga, Mohamad Fhaizal Mohamad Bukhori, Badrul Munir Md-Zain

**Affiliations:** 1 Centre for Pre-University Studies, Universiti Malaysia Sarawak, 94300, Kota Samarahan, Malaysia Centre for Pre-University Studies, Universiti Malaysia Sarawak, 94300 Kota Samarahan Malaysia; 2 Animal Resource Science and Management, Faculty of Resource Science and Technology, Universiti Malaysia Sarawak, 94300, Kota Samarahan, Malaysia Animal Resource Science and Management, Faculty of Resource Science and Technology, Universiti Malaysia Sarawak, 94300 Kota Samarahan Malaysia; 3 Department of Biological Sciences and Biotechnology, Faculty of Science and Technology, Universiti Kebangsaan Malaysia, 43600, Bangi, Malaysia Department of Biological Sciences and Biotechnology, Faculty of Science and Technology, Universiti Kebangsaan Malaysia, 43600 Bangi Malaysia

**Keywords:** metabarcoding, next-generation sequencing, primates, colobines, Borneo

## Abstract

The black morph Bornean banded langur (*Presbytischrysomelaschrysomelas*) is a critically endangered primate endemic to Sarawak, Borneo. Detailed knowledge of its diet is essential for guiding effective conservation strategies. This study employed DNA metabarcoding, targeting the chloroplast *trnL* region to characterise the dietary composition of *P.c.chrysomelas*. Faecal DNA from three individuals was extracted, amplified and sequenced using next-generation sequencing. Results identified 28 unique amplicon sequence variants corresponding to nine plant species spanning nine genera and eight families. The most abundant plant families were Euphorbiaceae and Achariaceae, with *Elateriospermum* and *Hydnocarpus* as dominant genera. At the species level, *Elateriospermumtapos* and *Hydnocarpusanthelminthicus* were the primary food sources. This study represents the first DNA metabarcoding-based dietary analysis of *P.c.chrysomelas*, providing valuable insights into its feeding ecology. These findings will support targeted conservation efforts, particularly within Tanjung Datu National Park, Sarawak.

## Introduction

The Bornean banded langur belongs to the Colobinae subfamily, a group of folivorous primates distinguished by their multi-chambered stomachs and fore-gut fermentation, adaptations that aid in digesting a leaf-based diet (Kirkpatrick 2011). Colobinae primates depend on specialised gut microbiota to facilitate the breakdown of fibrous plant material and to extract essential nutrients such as protein and vitamins (Liu et al. 2022). This digestive specialisation is essential for their survival in forest habitats.

Endemic to Borneo, the Bornean banded langur, *Presbytischrysomelaschrysomelas* is found exclusively in Malaysia, specifically within the State of Sarawak. Its distribution has been recorded in Tanjung Datu National Park (TDNP), Samunsam Wildlife Sanctuary (SWS), Similajau National Park (SNP), Gunung Pueh National Park (GPNP) and Maludam National Park (MNP) (Noor-Faezah et al. 2023, Nur-Aizatul et al. 2024, Nur-Aizatul et al. 2025). Despite its ecological significance in maintaining forest ecosystem dynamics, the specific dietary composition of the Bornean banded langur remains insufficiently documented. Understanding the dietary habits of primates is fundamental to conservation, offering critical insights for habitat management and species protection (Izar et al. 2025). This knowledge is particularly vital for species facing imminent extinction.

Previous dietary study has been done on *P.c.chrysomelas* in SWS from observational approaches, providing one of the earliest insights into its feeding ecology (Ampeng 2007). While informative, traditional approaches, such as direct observation and faecal fragment analysis, are hindered by observer bias and difficulties in accurately identifying plant material (Brun et al. 2022). However, recent advancements in molecular techniques have greatly enhanced the ability to assess wildlife diets. Amongst these, DNA metabarcoding via next-generation sequencing (NGS) has emerged as a powerful tool in ecological studies (Nor Hafisa Syafina et al. 2022, Khairulmunir et al. 2023, Mohd-Ridwan et al. 2024, Othman et al. 2024, Tingga et al. 2024a), including primate dietary research (Osman et al. 2020, Brun et al. 2022, Tingga et al. 2024b).

As a complementary approach to conventional methods, NGS enables the precise identification of plant taxa, including those that are morphologically indistinguishable or unidentifiable through field observations. Crucially, it utilises non-invasive samples such as faeces, allowing for dietary analysis without disturbing the animal’s natural behaviour (Beja-Pereira et al. 2009). Amongst the commonly used chloroplast DNA barcoding markers, *trnL* has demonstrated superior performance compared to rbcL, producing more sequence reads, offering higher taxonomic resolution and identifying a broader range of plant families (Mallott et al. 2018). Thus, in this study, we investigated the dietary composition of *P.c.chrysomelas* using DNA metabarcoding.

## Material and methods

### Study Site

**Figure 1.**
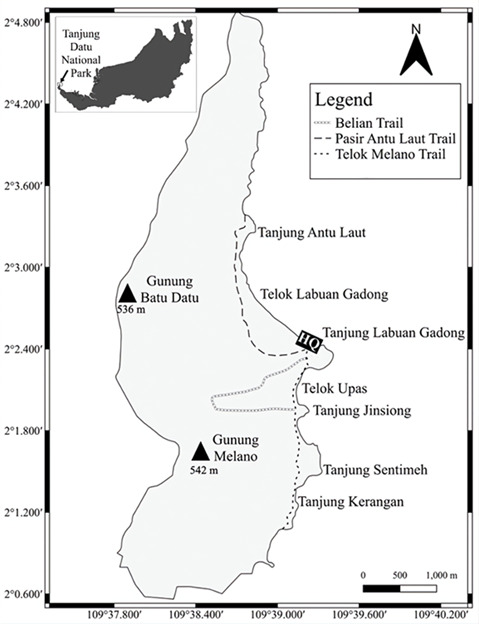
Map of Tanjung Datu National Park (TDNP).

Tanjung Datu National Park (TDNP) is one of the smallest national parks in Sarawak (2°03’19.48” N, 109°38’31.85” E), located at the south-western tip of the State (Fig. 1). Covering an area of 1,349 hectares, TDNP is renowned for its pristine ecosystems and high biodiversity. The Park features a unique combination of habitats, including primary mixed dipterocarp, kerangas, coastal, mangrove and sub-montane forests. Notably, it lies along the border between Malaysian Sarawak and Indonesian Kalimantan. In addition to being a well-known nesting site for sea turtles, TDNP supports diverse primate populations. These include the long-tailed macaque (*Macacafascicularis*), pig-tailed macaque (*Macacanemestrina*), silvered langur (*Trachypithecuscristatus*), Abbot’s gibbon (*Hylobatesabbotti*) and the focal species of this study, the Bornean banded langur (*Presbytischrysomelas*) (Mohd-Azlan et al. 2018, Nur-Aizatul et al. 2024, Nur-Aizatul et al. 2025).

### Sample Collection

**Figure 2.**
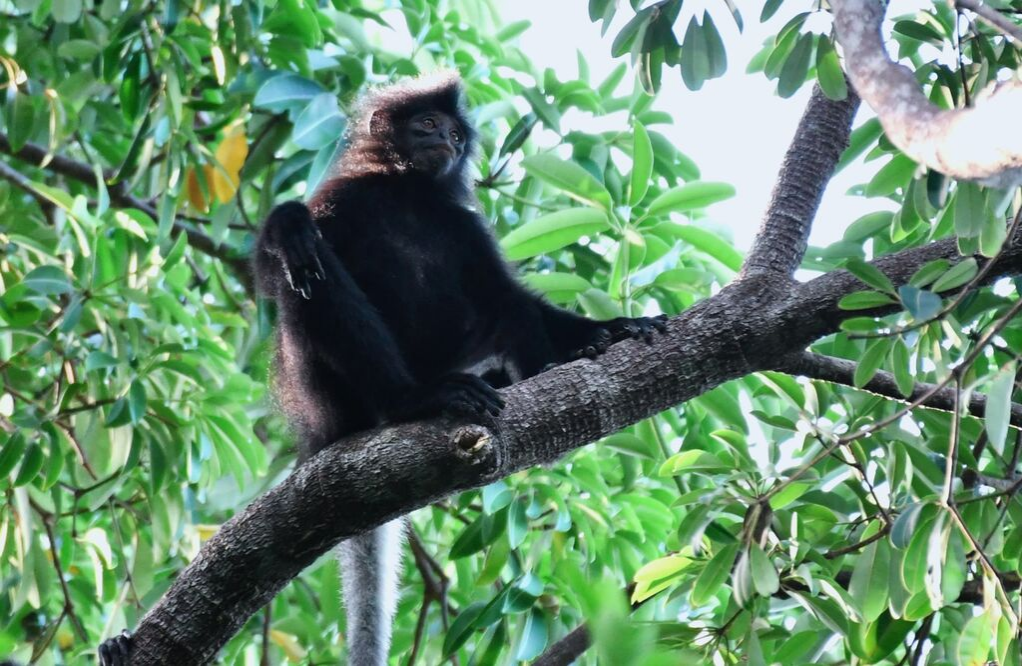
The black morph Bornean banded langur resting on a tree branch.

Non-invasive faecal sampling was conducted during surveys in TDNP from July 2023 to July 2024. During sampling, individual or groups of the Bornean banded langurs (Fig. 2) were followed and tracked throughout the day whenever possible. Alternatively, vocalisations were used to aid in tracking, particularly their distinctive "tat-tat-tat-tat" call (Ampeng et al. 2024). Three faecal samples were collected immediately after the langurs had retreated from the area. As the samples were collected opportunistically, information regarding the individual identity, age and sex of the animals was not available. To prevent human contamination, sterilised latex gloves and plastic spoons were used during collection. Each sample was labelled TD16, TD17 and TD24 and stored in a 50 ml centrifuge tube containing 25 ml of 95% ethanol. Samples were then refrigerated at −20°C in the Biology Research Laboratory, Centre for Pre-University Studies, Universiti Malaysia Sarawak.

### Laboratory procedures

Total genomic DNA was extracted from approximately 200 mg of faecal material using the Macherey-Nagel Stool DNA Kit, following the manufacturer’s recommended protocol. Polymerase chain reaction (PCR) was then performed using *trnL* primers targeting the P6 loop region of the chloroplast genome. Amplification was carried out using the forward primer *gtrnL* (5′-TCG TCG GCA GCG TCA GAT GTG TAT AAG AGA CAG GGG CAA TCC TGA GCC AA-3′) and the reverse primer *htrnL* (5′-GTC TCG TGG GCT CGG AGA TGT GTA TAA GAG ACA GCC ATT GAG TCT CTG CAC CTA TC-3′). The PCR reaction was conducted in a total volume of 25 μl, comprising 12.5 μl of REDTaq DNA polymerase, 1 μl each of the *gtrnL* and *htrnL* primers, 7.5 μl of deionised-distilled water (ddH₂O) and 3 μl of extracted DNA. Amplification reactions were performed in a Mastercycler thermal cycler, following the protocol described by Mohd-Ridwan (2021). The resulting PCR products were sent to Apical Scientific Sdn. Bhd. for sequencing. Sequencing was conducted using the Illumina MiSeq platform, employing 150 base pair (bp) paired-end (PE) reads.

### Data Analysis

The Amplicon sequence variants (ASVs) were constructed using the *gtrnL* and *htrnL* primers with overhang adapters via *trnL* (P6 loop) DNA metabarcoding (Taberlet et al. 2007). The resulting ASV data were then loaded into RStudio version 2024.04.2+764 (Posit team 2025) with the DADA2 packages (Callahan et al. 2016) for dietary characterisation and statistical analysis. The DADA2 was employed as the primary bioinformatics tool to process the sequencing data and correct errors in amplicon sequence variants (ASVs). The DADA2 workflow included quality assessment using FastQC, processing and merging of PE reads, chimera removal, taxonomic assignment and phylogenetic analysis. The identified plant taxa were checked against the preliminary report by Tawan et al. (2015) to confirm their presence in the study area. The taxonomic classification of ASVs was performed using the trnL plant reference database, compiled from GenBank sequences of plant species.

## Results

### Plant Taxa Identified from Faecal Samples of the Bornean Banded Langur

**Figure 3.**
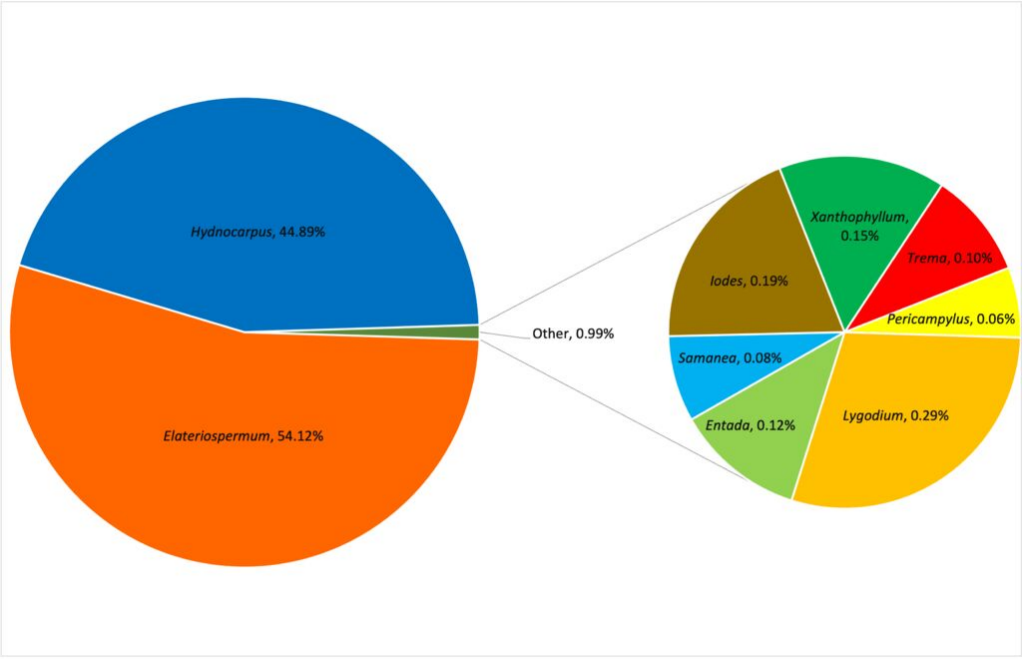
Relative abundance pie chart of the identified plant consumed by the *P.c.chrysomelas* at genus level in TDNP.

The *trnL* next-generation sequencing of three faecal samples yielded a total of 594,955 raw reads, which were reduced to 229,198 non-chimeric reads following error correction and chimera removal. These reads were clustered into 28 amplicon sequence variants (ASVs) used for dietary profiling. The diet of *P.c.chrysomelas* comprised nine plant species, spanning nine genera and eight families (Table 1). The most frequently consumed plants belonged to the family Euphorbiaceae, specifically the genus *Elateriospermum*, which contributed the highest relative abundance. This was followed by the family Achariaceae, represented by the genus *Hydnocarpus*. Other families and genera identified in the diet included Lygodiaceae (*Lygodium*), Fabaceae (*Entada*, *Samanea*), Icacinaceae (*Iodes*), Polygalaceae (*Xanthophyllum*), Cannabaceae (*Trema*) and Menispermaceae (*Pericampylus*) (Fig. 3). At the species level, *Elateriospermumtapos* constituted the most of the consumed plant material, followed by *Hydnocarpusanthelminthicus*. Other species, such as *Lygodiumcircinatum*, *Iodescirrhosa*, *Xanthophyllumaffine*, *Entadaphaseoloides*, *Tremaorientalis*, *Samaneasaman* and *Pericampylusglaucus*, were present in minor proportions (Table 1).

## Discussion

Understanding the dietary ecology of the Bornean banded langur is critical for providing information for conservation management, particularly within heterogeneous habitats such as TDNP. Therefore, this study is the first that employs the DNA metabarcoding technique to unveil the diet of the Bornean banded langur. Approximately 54.74% of the nine plant species identified were composed of species such as *E.tapos*, *L.circinnatum*, *X.affine*, *P.glaucus*, *I.cirrhosa* and *E.phaseoloides*, which are characteristic components of lowland dipterocarp forests (Cicuzza 2020, Jana and Jusoh 2021). In contrast, species such as *T.orientalis* and *S.saman*, which are commonly associated with disturbed or secondary forest environments (Durr 2001, Appau et al. 2024), reflecting their role as pioneer species, accounted for a small portion of the diet. Additionally, taxa, such as *E.phaseoloides*, *P.glaucus* and *T.orientalis*, are frequently found in riverine zones. The dietary diversity of *P.c.chrysomelas* reflects the spatial complexity of its forest environment. While it mainly consumes shade-tolerant, late-succession species in mature dipterocarp forests, its opportunistic feeding on pioneer and transitional plants in riverine suggests flexible foraging across different successional habitats.

Interestingly, none of the plant species recorded by Tawan et al. (2015) was detected in the diet of the Bornean banded langur at TDNP. Likewise, the taxa identified through metabarcoding were not present in their records. This absence of overlap may be due to differences in methodology, sampling period or the limited taxonomic scope of the previous survey. The integration of observational and molecular approaches provides a better understanding of the langur’s diet. To improve the accuracy of plant identification in future studies, the development of a comprehensive plant reference database for TDNP is recommended.

The dominance of *E.tapos* and *H.anthelminthicus* in the diet of the Bornean banded langur in TDNP indicates that edible seeds were abundant in the forest during the sampling period. The *E.tapos* has been documented as a crucial protein source for primates inhabiting resource-limited environments (McConkey and Chivers 2004). Similarly, *H.anthelminthicus* is known for its edibility and medicinal properties (Milow et al. 2014). Alongside these seeds, *P.c.chrysomelas* also consumes young leaves from species *L.circinnatum* and *E.phaseoloides*, in addition to fruits and flower species *X.affine*, *T.orientalis*, *P.glaucus* and *I.cirrhosa*. These young leaves generally contain less fibre, are more digestible and richer in protein than mature leaves (Matsuda et al. 2017). However, according to Hanya and Bernard (2015), seeds are better as an energy source due to an increase in lipid content, while young leaves showed no nutritional advantage over seeds. This implies that seeds are a better diet than young leaves if langurs merely consume them, based on availability and without selection. Other than benefitting the wildlife, it is interesting to note that certain plant species, such as *L.circinnatum* and *T.orientalis*, also have medicinal and cultural values amongst the local communities. *Lygodiumcircinnatum* is commonly used by the Iban and Lun Bawang communities, while *T.orientalis* is traditionally utilised by the Kendayan in preparing a tonic drink or ‘*air masak*’ (Paul 2000). These traditional uses of plant species illustrate significant parallels between wildlife ecology and ethnobotanical knowledge.

This preference for seeds and young leaves is notably consistent across populations of *P.c.chrysomelas* in Sarawak. In SWS, *P.c.chrysomelas* mainly consume young leaves of genera *Shorea*, Sapotaceae sp. and *Liana*, comprising 51.2% of its diet (Ampeng 2007). Although the plant taxa consumed vary according to habitat and seasonal variety, the plant parts being ingested, which are young leaves, fruits and seeds, remain consistent. Ampeng's findings corroborate the hypothesis that *P.c.chrysomelas* across Sarawak rely significantly on young leaves, complemented with periodic intake of *Baccaurea* sp. seeds (~ 1.9%) and fruits of Lauraceae, Dilleniaceae and *Eugenia* (~ 11.0%) depending on the seasonality. Moreover, no interspecies competition was found throughout his study period, suggesting that seed consumption may act as a method to reduce competition. In Jemoreng, the diet subspecies *P.c.cruciger* consists predominantly of fruiting plants like *Ficustinctoria*, *Pometiasuaveolens* and *Lansiumchinensis* (Tingga et al. 2024b). Additionally, both *P.c.cruciger* in Danau Sentarum National Park (Santoso et al. 2023) and *P.c.chrysomelas* in TDNP reportedly consumed the leaves of a common plant species, *X.affine*, suggesting a degree of shared dietary preference across subspecies and geographical regions. The recurrence of several taxa across populations may indicate frequent consumption of food sources in response to food availability amongst these langurs, even though the current study only provides a snapshot in time and excludes seasonal monitoring.

Future research should increase the faecal sample size from TDNP to enhance the reliability of dietary data. The current small sample size (n = 3) reflects challenges in locating faeces due to the species’ elusive behaviour and dense forest conditions. It is essential to collect samples year-round and across the species' home range. Additionally, future studies should consider the phenology and availability of local plant species, complemented by DNA barcoding to assess their abundance in faecal samples. Seasonal factors, such as fruiting, leaf flushing and dry-wet cycles, should be considered, as they likely influence feeding patterns. Rainfall variations also affect habitat carrying capacity by regulating plant growth and mammal breeding cycles. Finally, expanding the local plant DNA reference database is essential to improve species identification accuracy in dietary studies across Malaysia.

## Conclusions

This study provides preliminary dietary characterisation of the black morph Bornean banded langur through DNA metabarcoding analysis of three faecal samples from TDNP. A total of 28 amplicon sequence variants (ASVs) were detected, with nine resolved to species level and 19 remaining unclassified. The diet was dominated by *E.tapos* and *H.anthelminthicus*, with seven additional plant taxa present in lower relative abundance. Conservation management efforts should incorporate the plant species identified to support habitat preservation and species viability. These findings provide critical baseline data to provide information for conservation policies and habitat management for the Bornean banded langur.

## Figures and Tables

**Table 1.** Plant species consumed by *P.c.chrysomelas*, showing relative abundance (RA), succession stage (SS) and habitat type (HT).

**No.**	**Family**	**RA (%)**	**SS**	**HT**
	Species			
	**Euphorbiaceae**			
1.	*Elasteriospermumtapos* Blume (1826)	54.12	Late Succession	Lowland dipterocarp forest
	**Achariaceae**			
2.	*Hydnocarpusanthelminthicus* Pierre ex Gagnep (1908)	44.89	Late Succession	Lowland dipterocarp forest
	**Lygodiaceae**			
3.	*Lygodiumcircinatum* (Burm.) Sw. (1806)	0.29	Late Succession	Lowland dipterocarp forest
	**Fabaceae**			
4.	*Entadaphaseoloides* (L.) Merr. (1904)	0.12	Transitional Zone	Riverine forests
5.	*Samaneasaman* (Jacq.) Merr. (1914)	0.08	Pioneer Stage	Secondary forest
	**Icacinaceae**			
6.	*Iodescirrhosa* Turcz. (1855)	0.19	Late Succession	Lowland dipterocarp forest
	**Polygalaceae**			
7.	*Xanthophyllumaffine* Korth. ex Miq. (1864)	0.15	Late Succession	Lowland dipterocarp forest
	**Cannabaceae**			
8.	*Tremaorientalis* Blume (1852)	0.1	Pioneer Stage	Secondary forest
	**Menispermaceae**			
9.	*Pericampylusglaucus* (Lam.) Merr.(1917)	0.06	Transitional Zone	Riverine forests
